# Comparative Efficacy of Bulevirtide, Interferons, and Nucleos(t)ide Analogs for Chronic Hepatitis Delta: A Systematic Review and Network Meta‐Analysis

**DOI:** 10.1155/ijh/6325996

**Published:** 2026-03-26

**Authors:** Rohan Karkra, Mahinaz Mohsen, Joshua E. Pagán-Busigó, Ethan Shamsian, Michael Bebawy, Steven Rella, Zaid Tafesh, Paul Gaglio

**Affiliations:** ^1^ Department of Medicine, Rutgers New Jersey Medical School, Newark, New Jersey, USA, rutgers.edu; ^2^ Department of Medicine, Division of Liver Disease & Transplant Hepatology, Rutgers New Jersey Medical School, Newark, New Jersey, USA, rutgers.edu

**Keywords:** bulevirtide, chronic hepatitis, hepatitis D, peginterferon alpha, viral hepatitis

## Abstract

**Background:**

Chronic hepatitis D virus (HDV) continues to be a global health concern, and the infection remains challenging to treat. Over the past few decades, pegylated interferons, typically in combination with therapy for hepatitis B, have become the standard of care, with considerable side effects and frequently unsatisfactory results. Several new therapies are emerging, including bulevirtide and lonafarnib. We conducted a systematic review and network meta‐analysis (NMA) to compare the efficacy of bulevirtide, interferons, and nucleos(t)ide analogs, alone or in combination, for the treatment of chronic hepatitis D.

**Methods:**

PubMed, Embase, and Cochrane databases were searched for randomized controlled trials and nonrandomized interventional studies assessing HDV RNA suppression, biochemical response, combined response, and histological improvement with various therapies. NMA was performed using a frequentist random‐effects model. Odds ratios (OR) with 95% confidence intervals (CI) were calculated and forest plots were generated.

**Results:**

Thirteen studies (*n* = 922) were included, studying nine possible treatment arms. At the end of treatment, 31.8% achieved a virological response. Bulevirtide monotherapy (OR 38.63, *p* < 0.05) and combinations with NA (OR 69.36, *p* < 0.05) and peginterferon alpha (OR 260.08, *p* < 0.05) significantly suppressed HDV RNA, with the bulevirtide–peginterferon alpha combination having the highest HDV RNA suppression. At ≥ 24‐week follow‐up, no regimen maintained significant HDV RNA suppression. Biochemical response was achieved in 42.7% at the end of treatment; however, no group reached statistical significance versus control, though bulevirtide and its combination with peginterferon alpha outperformed peginterferon alpha alone. Combined response was highest with bulevirtide–peginterferon alpha (OR 112.69, *p* < 0.05), followed by bulevirtide monotherapy. Histological response (five studies, *n* = 183) was not statistically significant with any intervention compared with control.

**Conclusion:**

Bulevirtide and peginterferon alpha combination therapy may offer the most promising treatment for chronic hepatitis D. More studies are needed to assess the efficacy of this regimen and establish the optimum dose and duration of treatment.

## 1. Introduction

Hepatitis D virus (HDV) is a pathogenic human virus that requires hepatitis B virus (HBV) for replication [1]. Its envelope, composed of hepatitis B surface antigen (HBsAg), mediates hepatocyte entry via the sodium taurocholate cotransporting polypeptide (NTCP) receptor [2]. HDV transmission occurs via two main pathways: coinfection, in which HBV and HDV are acquired simultaneously, and superinfection, where HDV infects individuals with pre‐existing chronic HBV infection. Although coinfection often resolves spontaneously in immunocompetent patients, it may result in severe acute hepatitis or, rarely, fulminant liver failure. Superinfection more frequently progresses to chronic HDV infection, markedly increasing the risk of cirrhosis, hepatic decompensation, and hepatocellular carcinoma [1].

HDV is primarily transmitted through parenteral exposure, particularly among people who inject drugs. Additional routes include exposure to unscreened blood products, unsafe medical or cosmetic practices, and, less commonly, vertical or sexual transmission. Nosocomial outbreaks have been reported following lapses in infection control [3]. Global prevalence is estimated at 12–72 million, with the highest burden in Eastern Europe, Central Asia, the Middle East, Western and Central Africa, and parts of South America. In high‐income countries, HBV vaccination programs have shifted HDV prevalence toward high‐risk groups, particularly individuals with HIV or HCV coinfection, men who have sex with men, sex workers, and people who inject drugs [4].

For decades, pegylated interferon alpha was the only available treatment, offering modest virological response rates (20%–30%), high relapse rates, and substantial adverse effects, including cytopenias and neuropsychiatric symptoms, limiting use in advanced liver disease. Pegylated interferon lambda (PEG‐IFN*λ*), which selectively targets hepatocytes, has shown improved tolerability but raised safety concerns [3, 5].

Bulevirtide (BLV), a first‐in‐class NTCP entry inhibitor developed by Gilead, received conditional European Medicines Agency (EMA) approval in 2020 as the first targeted therapy for HDV. By mimicking the preS1 domain of HBsAg, it competitively binds NTCP, preventing reinfection of hepatocytes. Clinical trials (MYR201–204, MYR301) and real‐world studies demonstrate its efficacy in reducing HDV RNA, improving liver biochemistry, and enhancing quality of life, with a favorable safety profile across disease stages, including decompensated cirrhosis. It was notably denied approval by the FDA in the United States, with the decision letter citing manufacturing issues, rather than efficacy or safety concerns [6, 7]. Gilead sciences is expected to submit another application to the FDA soon.

Other agents in development include lonafarnib, a prenylation inhibitor that disrupts virion assembly, particularly in combination with ritonavir and PEG‐IFN*α*; monoclonal antibodies such as HH003 and tobevibart (VIR‐3434) targeting the preS1 domain or HBsAg to block viral entry; siRNA agents like JNJ‐3989 to reduce HBsAg expression; and nucleic acid polymers (e.g., REP 2139) that inhibit particle assembly [8].

Despite these advances, several challenges remain as follows: prolonged treatment courses, frequent relapse after discontinuation, intolerable side effects, and limited options for decompensated patients. Real‐world evidence is needed to clarify optimal treatment duration, maintenance strategies, and early intervention benefits. This systematic review and network meta‐analysis is necessary to study the available data on the efficacy of therapies for chronic HDV infection and objectively assess various treatment approaches.

## 2. Methods

### 2.1. Search Strategy and Protocol

We conducted a comprehensive search of PubMed, Cochrane, and Embase databases from inception to date (March 2025) to identify all studies studying the effectiveness of an intervention in the management of chronic hepatitis D. We utilized a search strategy as follows: (“Hepatitis D”[Mesh] OR “Hepatitis delta virus”[tiab] OR “HDV”[tiab] OR “Delta agent”[tiab]) AND (“bulevirtide”[tiab] OR “Myrcludex B”[tiab] OR “Hepcludex”[tiab] OR “interferon”[tiab] OR “peginterferon”[tiab] OR “pegylated interferon”[tiab] OR “nucleoside analog”[tiab] OR “nucleotide analog”[tiab] OR “NA therapy”[tiab] OR “tenofovir”[tiab] OR “entecavir”[tiab] OR “lamivudine”[tiab]) and filtered for randomized control trials and interventional studies. We manually reviewed the references to find other suitable studies that may have been missed. The search terms were adjusted as needed for compatibility with the respective databases. This review conformed to the Preferred Reporting Items for Systematic reviews and Meta‐Analyses (PRISMA) [9] guidelines. The review was registered on PROSPERO—CRD420251107282.

### 2.2. Study Selection

Three authors (R.K., M.M., and J.E.P‐B.) screened all the identified studies. The following criteria were required for inclusion: [1] The studies were randomized controlled trials or interventional studies; [2] the study population included patients with chronic hepatitis D (detectable HDV‐RNA levels and HDV antibodies in serum at least 6 months before the study), who were followed for at least 24 weeks after treatment initiation with different regimens; [3] an antiviral agent such as interferon, pegylated interferon, nucleos(t)ide analog (NA) or BLV, or a combination of multiple agents was included as an intervention group; [4] complete HDV suppression and/or ≥ 2 log decrease in HDV RNA in serum, biochemical response, and histological response at the end of treatment (EOT) and/or at ≥ 24 weeks of follow‐up were recorded. (For the purposes of this review, biochemical response is defined as the normalization of serum ALT (≤ 1 × ULN) and histological response is defined as a ≥ 2 decrease of the Knodell score or a ≥ 1 decrease of the Ishak score); [5] there were at least two distinct intervention groups, including placebo; and [6] the study was in English. Any study that did not have at least two treatment arms (including placebo/no treatment) was excluded, as this study design is incompatible with a network meta‐analysis without introducing bias. All studies published in a language other than English and those that could not be retrieved were excluded. The final number of studies identified, screened, rejected, and included is shown below on the PRISMA chart.

### 2.3. Data Collection

Although we identified several drugs in the literature that have been studied in the management of hepatitis D, some of them, such as lonafarnib, could not be included in this review. This was because the study designs were incompatible with our inclusion criteria and/or did not fit the network meta‐analysis model. Additionally, due to a lack of abundant data and few eligible studies, the authors decided to study therapeutic classes. Therefore, the interventions were grouped into categories: BLV, interferons, pegylated interferons, and NA (including lamivudine, tenofovir, and entecavir). The various interventions were placed into their respective categories regardless of dose. Four authors independently (R.K., M.M., J.E.P‐B., and M.B.) extracted prespecified characteristics from all included studies, such as study design, country of study, number of subjects, their baseline characteristics, interventions, duration of treatment and follow‐up, ALT normalization, ≥ 2 log decrease in HDV RNA, histological improvement, and HDV undetectability.

### 2.4. Quality Assessment

Two authors (S.R. and E.S.) independently evaluated the quality of the included studies, using the Cochrane Risk of Bias Assessment Tool (RoB 2.0) for randomized control trials [10], incorporating six items: bias in the random allocation method, bias in allocation concealment, bias in blinding, bias in data integrity, bias with or without selective reporting, and other sources of risk of bias. A third member (R.K.) made the final decision if there was a discrepancy between the two reviewers.

### 2.5. Statistical Analysis

We performed a network meta‐analysis, using a frequentist random effects model, which produces a network of direct and indirect comparisons between the outcomes of different treatment interventions. We utilized MetaInsight, a statistical software funded and hosted by the National Institute for Health and Care Research (NIHCR) [11]. MetaInsight is part of the Complex Reviews Synthesis Unit (CRSU) suite of evidence synthesis apps. It utilizes the R package “netmeta” for frequentist models and calculations. The studied endpoints included complete HDV RNA suppression, ALT normalization, and combined treatment response, defined as ≥ 2 log decrease in HDV RNA or undetectable RNA, with ALT normalization, at the EOT and at follow‐up (≥ 24 weeks). Given that we looked at binary outcomes, we calculated odds ratios (OR). *p* < 0.05 was considered significant, and 95% confidence intervals were noted. Forest plots were generated to represent the results. *I* ^2^ was used to test for heterogeneity among studies, and inconsistency was assessed.

## 3. Results

### 3.1. Search Results

**Figure 1:**
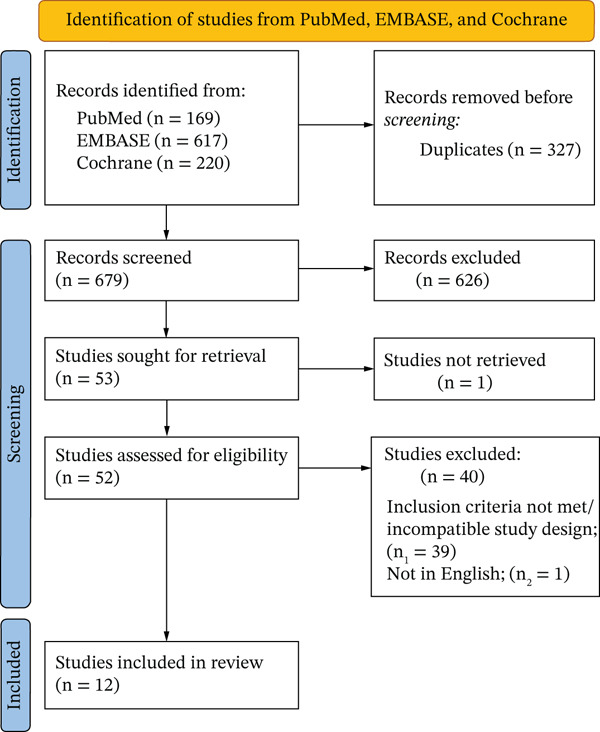
PRIMSA chart showing the schema of study selection.

After removing duplicates and preliminary screening, we identified 52 studies that were selected for further review. After full text review, a total of 12 studies were included in this network meta‐analysis, comprising 869 patients across nine treatment arms (BLV, interferon alpha, peginterferon alpha, NAs, and their combinations). The full schema of article selection is shown in the PRISMA flowchart in Figure 1 below.

### 3.2. Baseline Characteristics

We have provided a complete summarization of all studies included in Table 1 below. A total of 12 studies were included. Across all the studies, there were 619 males, with ages of participants ranging from 17 to 67. Most studies were multicentric and covered sites across multiple countries and continents. Seven studies documented the history of prior interferon exposure. In the studies that did report on this, 42.2% of the patients had prior exposure. The treatment duration was at least 24 weeks and ranged from 24 weeks to 104 weeks. The follow‐up duration varied across studies, but 24 weeks (6 months) was used as the cut‐off time‐point in this analysis. In a study by Wedemeyer et al. [6], the treatment arms switched at 48 weeks. In this case, only data up to 48 weeks was included in our analysis. At the EOT, 12 studies assessed for biochemical response, 12 assessed for HDV RNA suppression, and 5 assessed for histological response. At follow‐up > 6 months after treatment, eight studies assessed for biochemical response, nine assessed for HDV RNA suppression, and none assessed for histological response.

**Table 1:** Summarization of all studies included in this review (IFN—interferon).

Author (year)	Study location	Interventions	Number of patients	*M* *e* *a* *n* *a* *g* *e* ± *S* *D*/*r* *a* *n* *g* *e*	Males (*n*)	Treatment duration (weeks)	Follow‐up duration (weeks)
Yurdaydin et al. [12]	Multicenter	Lamivudin IFN alpha 2a Lamivudin + IFN alpha 2a	39	38 (20–67)	33	52	26
Bogomolov et al. [13]	Single center	Bulevirtide Pegylated IFN alpha 2a + bulevirtide Pegylated IFN alpha 2a	23	Range: 23–61	17	24 ‐72	24
Canbakan et al. [14]	Single center	IFN alpha 2b IFN alpha 2b + lamivudine	26	43.83 ± 8.57, 42.5 ± 11.02, respectively	15	48	3.1 ± 1.9 years
Wedemeyer et al. [6]	Multicenter	Bulevirtide No treatment	150	42 ± 8.4	86	144 (Data studied at 48 weeks)	96
Asselah et al. [15]	Multicenter	Pegylated IFN alpha 2a Bulevirtide + pegylated IFN alpha 2a Bulevirtide	174	41	124	96	48
Niro et al. [16]	Multicenter	Pegylated IFN alpha 2b + ribavirin Pegylated IFN alpha 2b	38	44 ± 9.2	23	72	24
Wedemeyer et al. [17]	Multicenter	Bulevirtide + tenofovir Tenofovir	118	40.2 ± 9.5	79	24	48
Gunsar et al. [18]	Single center	IFN alpha 2a IFN alpha 2a + ribavirin	31	39 ± 9, 38 ± 11, respectively	20	104	24
Farci et al. [19]	Single center	IFN alpha 2a No treatment	42	33.7 (21–60)	33	48	24
Lampertico et al. [7]	Multicenter	Bulevirtide Bulevirtide + Pegylated IFN alpha 2a Bulevirtide + tenofovir	63	36 (18–62)	57	48	24
Rosina et al. [20]	Multicenter	IFN alpha 2a No treatment	60	Range: 18–65	44	48	48
Wedemeyer et al. [21]	Multicenter	Pegylated IFN alpha 2a Pegylated IFN alpha 2a + adefovir Adefovir	80	Median: 38, 42, and 33, respectively	56	48	24

### 3.3. Quality assessment

**Figure 2:**
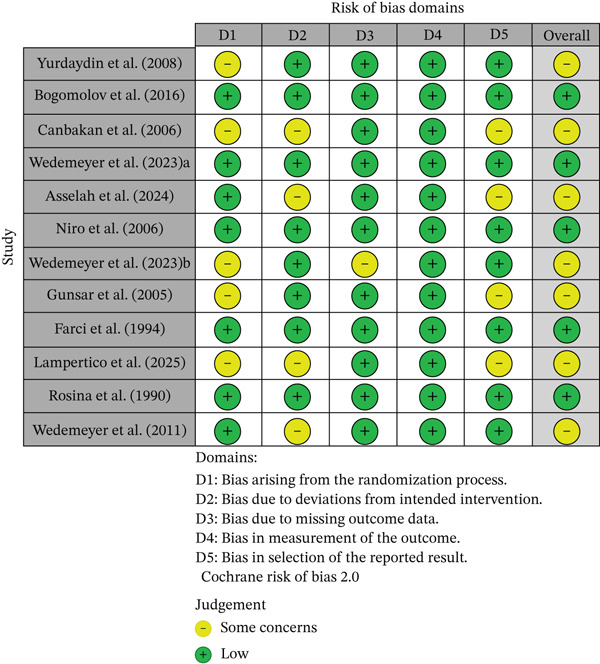
Risk of bias assessment.

The Cochrane risk of bias (RoB 2) was applied to all randomized studies. Five studies were deemed to have low risk, and seven studies had some concerns. The results of the bias assessment are provided in Figure 2 below.

### 3.4. Network Meta‐Analysis

#### 3.4.1. HDV RNA Suppression

**Figure 3:**
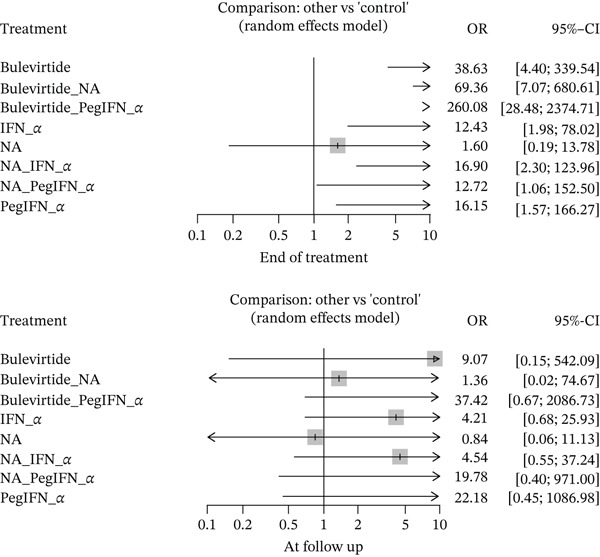
Forest plot depicting HDV RNA suppression.

At the EOT, 12 studies covering 869 patients reported on the undetectability of HDV RNA. The typical treatment duration ranged from 24 to 48 weeks. A total of 277/869 (31.8%) patients had undetectable HDV RNA. At follow‐up, nine studies assessed RNA levels. These included 576 patients, and 106 of them had persistent HDV RNA suppression (18.4%). The forest plots depicting the results are shown in Figure 3 below.

BLV, both as monotherapy (OR = 38.63, *p* < 0.05) and in combination with either NA (*OR* = 69.36, *p* < 0.05) or peginterferon alpha (OR = 260.08, *p* < 0.05), was able to suppress HDV RNA to undetectable levels. Interferon alpha (OR = 12.43, *p* < 0.05) and peginterferon alpha (*OR* = 16.15, *p* < 0.05) also achieved RNA suppression alone, and in combination with NA (OR = 16.9 and OR = 12.72, respectively, *p* < 0.05), but NA monotherapy was not able to achieve undetectable RNA levels (OR = 1.6, p > 0.05). A test for inconsistency was *p* > 0.05 for all treatment groups, indicating that there was no inconsistency in direct and indirect comparisons.

A summary plot indicated that BLV and peginterferon alpha combination had the highest rank and the highest odds of inducing HDV RNA suppression. This was followed by a BLV and NA combination and BLV monotherapy. All treatment groups ranked higher than control, with NA monotherapy performing the worst out of all treatments. The combination of BLV and peginterferon alpha, when compared with BLV alone, showed statistically significant RNA suppression (OR = 6.73, *p* < 0.05).

At follow‐up of > 24 weeks or 6 months, no treatment group had statistically significant persistent HDV RNA suppression. The odds of having HDV RNA suppression were as follows: BLV (9.07; 95% CI: 0.15, 542.09), BLV and peginterferon alpha (37.42; 95% CI: 0.67, 2086.73), BLV and NA (1.36; 95% CI: 0.02, 74.67), peginterferon alpha (22.18; 95% CI: 0.45, 1086.98), interferon alpha (4.2; 95% CI: 10.68, 25.93), NA (1.18; 95% CI: 0.09, 15.59), peginterferon alpha and NA (19.78; 95% CI: 0.40, 971.00), and interferon alpha with NA (4.54; 95% CI: 0.55. 37.24). The test for inconsistency was again *p* > 0.05 for all treatment groups, indicating that there was no inconsistency in direct and indirect comparisons.

Peginterferon alpha with BLV had higher odds of having HDV RNA suppression (defined as ≥ 2 log decrease in HDV RNA in serum or complete undetectability) than BLV monotherapy alone (4.13; 95% CI: 1.60, 10.62). BLV with peginterferon alpha (44.30; 95% CI: 2.02, 969.70), peginterferon alpha (26.25; 95% CI: 1.42, 484.80), and peginterferon alpha with NA (23.41; 95% CI: 1.26, 433.32) had higher odds of having HDV RNA suppression compared with NA alone. However, BLV alone did not have a statistically significant difference (10.74; 95% CI: 0.45, 257.11). The wide confidence intervals, however, raise some concerns about the strength of the findings.

#### 3.4.2. Biochemical Response

**Figure 4:**
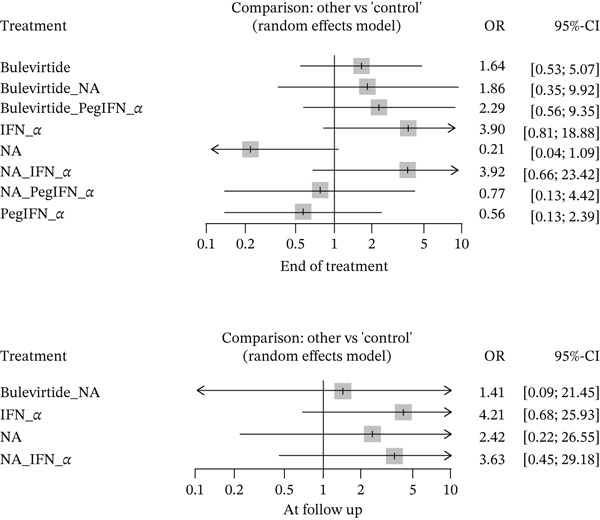
Forest plot depicting biochemical response.

At the EOT, 12 studies covering 869 patients reported on ALT normalization. A total of 371/869 (42.69%) patients across all the arms had ALT normalization. However, after running a network meta‐analysis, no treatment group showed statistically significant ALT normalization. At follow‐up, only eight studies had reported on ALT. However, two studies were disconnected from the network and hence could not be included in the analysis. The six studies included in the analysis had 304 patients among them, of which 50 (16.4%) had ALT normalization. The forest plots depicting the results are shown in Figure 4 below.

The odds of having a biochemical response were as follows: BLV (1.64; 95% CI: 0.53, 5.07), BLV and peginterferon alpha (2.29; 95% CI: 0.56, 9.35), BLV and NA (1.86; 95% CI: 0.35, 9.92), peginterferon alpha (0.56; 95% CI: 0.13, 2.29), interferon alpha (3.9; 95% CI: 0.81, 18.88), NA (0.21; 95% CI: 0.04, 1.09), peginterferon alpha and NA (0.77; 95% CI: 0.13, 4.42), and interferon alpha with NA (3.92; 95% CI: 0.66, 23.42). A test for inconsistency was *p* > 0.05 for all treatment groups, indicating that there was no inconsistency in direct and indirect comparisons.

Interferon alpha had a higher odds of ALT normalization compared with peginterferon alpha (7.02; 95% CI: 1.21, 40.84); however, it did not perform better than BLV (2.38; 95% CI: 0.44, 12.84) or BLV with peginterferon alpha (1.70; 95% CI: 0.28, 10.18). However, compared with peginterferon alpha, BLV (2.94; 95% CI: 1.05, 8.29) as well as its combination with peginterferon alpha (4.12; 95% CI: 1.52, 11.16) had higher odds of ALT normalization. NA monotherapy had lower odds of having ALT normalization than BLV, BLV with peginterferon alpha, BLV with NA, interferon alpha, and interferon alpha with NA (*p* < 0.05). NA monotherapy did not have higher odds of ALT normalization than peginterferon alpha (2.62; 95% CI: 0.67, 10.17).

At follow‐up, no group had statistically significantly increased odds of having ALT normalization compared with controls. Only four treatments were able to be assessed as part of the network plot—BLV with NA (1.41; 95% CI: 0.09, 21.45), interferon alpha (4.21; 95% CI: 0.68, 25.93), interferon alpha with NA (3.63; 95% CI: 0.45, 29.18), and NA (2.42; 95% CI: 0.22, 26.55).

#### 3.4.3. Combined Response

Combined response as described above refers to a combined biochemical and virological response, with virological response referring to both HDV RNA suppression and ≥ 2 log decrease in HDV RNA in serum.

**Figure 5:**
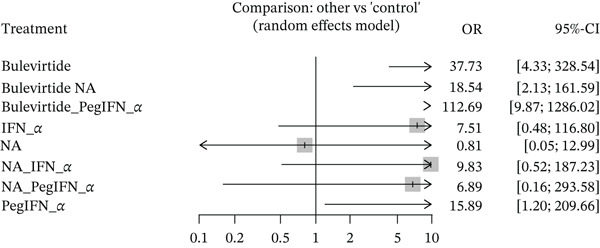
Forest plot depicting combined response at the end of treatment.

We found nine studies that reported data on combined response at the EOT, covering 731 patients. A total of 272 (37.2%) patients had a combined response at the EOT. BLV (37.73; 95% CI: 4.33, 328.54), BLV with pegylated interferon alpha (112.69; 95% CI: 9.87, 1286.02), BLV with NA (18.54; 95% CI: 2.13, 161.59), and pegylated interferon alpha alone (15.89; 95% CI: 1.20, 209.66) had statistically significant odds of having a combined response compared with controls. NA alone (0.81; 95% CI: 0.05, 12.99), with peginterferon alpha (6.89; 95% CI: 0.16, 293.58), and with interferon alpha (9.83; 95% CI: 0.52, 187.23) did not have statistically significant odds of a combined response. A test of inconsistency had a *p* > 0.05 for all treatments, indicating consistency between direct and indirect comparisons. The results are shown in Figure 5.

Upon ranking, BLV with pegylated interferon alpha had the highest odds of having a combined response at the EOT, followed by BLV and BLV with NA combination. Pegylated interferon alpha was ranked 4th out of all the treatments. In a pairwise meta‐analysis with BLV, the combination still had higher odds of combined response (4.17; 95% CI: 1.05, 16.50). BLV with pegylated interferon alpha also outperformed peginterferon alpha alone in a pairwise meta‐analysis (7.29; 95% CI: 1.45, 36.78).

The studies at follow‐up were disconnected and lacked the ability to make indirect comparisons. Therefore, the software could not analyze them under a random effects model. Therefore, we were unable to assess the efficacy of agents at follow‐up.

#### 3.4.4. Histological Response

Histological response was assessed in studies inconsistently and infrequently, likely due to complications associated with biopsies and difficulty obtaining consents for repeat procedures. We found that five studies assessed for histological response at the EOT. Among the 183 patients who had a biopsy, 73 (39.8%) showed a histologic response. The network model was able to look at direct and indirect comparisons between NA, interferon alpha with NA, interferon alpha, and BLV with NA. Although there was at least one study that also assessed peginterferon alpha, the model was unable to analyze this study due to a disconnect with the other studies, making it impossible to make an indirect comparison. However, no therapeutic intervention, alone or in combination, showed statistically significant improvement in liver histology compared with control (no intervention). We did not find studies during our analysis that assessed histological response at the end of follow‐up. Given the limited number of studies that documented histological response, the strength of this analysis is weak, and it is difficult to draw conclusions.

## 4. Discussion

This network meta‐analysis is aimed at comparing the efficacy of BLV, pegylated interferon, and NAs in the management of chronic HDV infection. The findings from the included studies demonstrate a marked difference in the efficacy of these treatments in the context of HDV RNA suppression, biochemical response, and histological improvement.

With regard to HDV RNA suppression, BLV+ pegylated interferon alpha showed the highest odds of achieving suppression at the EOT, with an OR of 260.08 (*p* < 0.05), far outperforming other regimens. BLV monotherapy (OR 38.63, *p* < 0.05) and its combinations with NAs (OR 69.36, *p* < 0.05) and pegylated interferon alpha (OR 260.08, *p* < 0.05) were significantly better at inducing RNA suppression compared with controls. However, despite these promising results during therapy, long‐term control remains elusive, as persistent HDV RNA suppression at follow‐up was minimal, with only 18.4% of patients maintaining undetectable RNA after more than 24 weeks of follow‐up. This highlights the transient nature of viral suppression with current therapies and underscores the need for maintenance strategies.

Interestingly, biochemical response was not statistically significant in any treatment regimen at the EOT or at follow‐up, despite some trends observed with combination therapies. At the EOT, 42.7% of patients achieved ALT normalization across all treatment arms, yet no regimen demonstrated a statistically significant improvement over control, demonstrating the limited impact of antiviral therapies on liver function normalization. BLV, both as monotherapy (OR 1.64; 95% CI: 0.53, 5.07) and in combination with pegylated interferon alpha (OR 2.29; 95% CI: 0.56, 9.35), showed trends toward improving biochemical response, but these differences did not reach statistical significance. In contrast, NA monotherapy, which performed the worst in terms of HDV RNA suppression, also demonstrated the lowest odds of achieving ALT normalization (OR 0.21; 95% CI: 0.04, 1.09), confirming what has been well known for decades that NAs alone are ineffective in improving liver function in chronic HDV.

Interpretation of ALT normalization in these studies requires consideration of the underlying virological phenotype. Most included patients had chronic HDV infection with either suppressed or low‐level HBV DNA, reflecting the well‐described suppressive effect of HDV on HBV replication [2]. In this context, hepatic inflammation and ALT elevation are predominantly HDV‐driven rather than HBV‐mediated. This distinction is important, as established HBV therapies such as NAs and pegylated interferon were evaluated in HBV mono‐infection, where biochemical activity directly reflects HBV replication. In contrast, therapies targeting viral entry or HDV replication may not yield comparable ALT responses if HBV activity is minimal at baseline. Therefore, the absence of significant differences in ALT normalization across treatment groups should be interpreted within this distinct virological framework.

Moreover, when assessing combined response, which includes both virological and biochemical improvements, the combination of BLV and pegylated interferon alpha demonstrated the highest odds of achieving a combined response at the EOT (OR 112.69; 95% CI: 9.87, 1286.02). This was followed by BLV monotherapy (OR 37.73; 95% CI: 4.33, 328.54), reflecting the potential benefit of combining antiviral agents to target both viral suppression and liver inflammation. No treatment regimen showed significant histological improvement, highlighting the gap between achieving virologic suppression and liver healing, although the authors advise caution in interpreting this, as there was insufficient data to make a powered analysis. Individual studies did show histological improvements with various therapies; however, our network meta‐analysis model was unable to identify those differences.

The findings of our study are largely consistent with recent studies comparing HDV treatment. BLV has emerged as a leading option in chronic HDV management, supported by real‐world data and clinical trials [15]. The major studies exploring the use of BLV monotherapy and in combination with pegylated interferon alpha are the MYR studies. Similar to the MYR‐201 [22] and MYR‐202 [17] trials, we observed a substantial reduction in HDV RNA, although biochemical response in terms of ALT normalization was not statistically significant. The MYR‐202 trial demonstrated dose‐dependent responses and a high relapse rate posttreatment, which is again seen in our analysis, which also found that although BLV monotherapy performed well in virologic suppression, it did not sustain long‐term benefits at follow‐up. Additionally, the MYR‐301 trial′s findings of sustained responses in 45%–48% of patients align with our observed outcomes, where BLV showed notable initial efficacy but did not demonstrate lasting biochemical improvement or histological response [6]. It is exciting to note that there is now evidence that BLV is safe in cirrhosis as shown by Dietz‐Fricke et al. [23], which allows these vulnerable patients to get treatment.

The combination of BLV and pegylated interferon, although showing promising HDV suppression, did not reach statistical significance in regard to ALT normalization. This is in contrast to the MYR studies, which revealed positive results in terms of HDV suppression and biochemical response [6, 17, 22]. In MYR‐201, 5 of 7 patients achieved undetectable HDV RNA with combination therapy. MYR‐203 and MYR‐204 [15] demonstrated enhanced HDV RNA suppression, improved biochemical markers, and higher rates of sustained virologic response compared with monotherapy. The role of monotherapy with pegylated interferon in the management of chronic HDV remains controversial. Although it continues to be considered a standard therapeutic option at present, our study reaffirms the modest success rates of interferon alpha (OR = 12.43) and peginterferon alpha (OR = 16.15) monotherapy in achieving HDV RNA suppression, indicating that IFN should be used in combination with BLV unless there is a contraindication. Previous studies have also noted high relapse rates with pegylated interferon‐alpha treatments, which is consistent with our findings of limited sustained responses posttreatment.

The findings of our meta‐analysis have several clinical implications. First, they confirm what hepatologists have known about the efficacy of NAs like tenofovir and entecavir in the context of viral hepatitis. It is now well established that NA monotherapy does not have a role in hepatitis D management [24]. However, since previous studies have evaluated NAs in the context of hepatitis D infection, including them provided the necessary common nodes to enable comparisons. The consistent reduction in HDV RNA levels observed across the included studies suggests that the tested therapies are biologically active and can potentially translate into improved clinical outcomes if sustained over time. Combining BLV and pegylated interferon alpha has demonstrated higher rates of undetectable HDV RNA compared with BLV monotherapy in our analysis, with OR exceeding 250, and may become the preferred initial therapy in select patients. Our results suggest that combination therapy may be beneficial for patients in whom rapid viral suppression is needed, such as those with mild to moderate fibrosis.

As mentioned earlier, BLV is an entry inhibitor. Although it can prevent de novo viral entry into hepatocytes, it is unable to target intracellular viral particles. Therefore, lapse in benefits of therapy upon treatment discontinuation is biologically plausible [22]. This limitation also extends to hepatitis B. Although it has demonstrated efficacy in chronic hepatitis D, BLV monotherapy has not shown significant reductions in HBsAg levels, and available data are limited. Because the drug prevents new viral entry without affecting established infection or viral replication from existing covalently closed circular DNA (cccDNA), its utility as a standalone therapy for chronic hepatitis B is limited [17]. Similarly, NAs, which specifically inhibit the reverse transcription process of the HBV, do not directly reduce surface antigen levels and based on their mechanism of action, are not expected to exert a meaningful antiviral effect against HDV [12]. In contrast, combination therapy with interferon, which has antiviral activity against both RNA and DNA viruses, may reasonably enhance the efficacy of BLV [18].

However, BLV monotherapy, which is currently one of the first‐line therapies for chronic hepatitis D infection, can remain as an effective alternative option, especially for patients with contraindications to interferon, such as those with advanced fibrosis and/or portal hypertension, as it has demonstrated improvement in response rates with longer treatment durations.

Despite combination therapy and monotherapy demonstrating positive outcomes with regard to HDV RNA suppression, it is uncertain whether that also translates into histological improvement, as it was rarely assessed, with only five studies including paired biopsies. These findings suggest that although viral suppression is achievable, liver injury persists in some patients. Therefore, until long‐term outcomes, such as fibrosis regression, hepatic decompensation, and HCC incidence, are well ascertained, patients should continue routine surveillance even if they achieve virologic response with these medications, as the impact of these therapies on biochemical response and histological improvement remains suboptimal [25].

Several limitations of the current evidence and trials must be acknowledged. There is marked heterogeneity between studies, which complicates the interpretation of pooled outcomes and limits their generalizability. Dosing regimens varied between studies, with BLV evaluated at 2, 5, and 10 mg daily. There was also variation in baseline patient characteristics with respect to fibrosis stage and HBV control level. Such variability likely contributed to the wide confidence intervals observed in our network meta‐analysis and underscores the need for standardized trial designs that stratify by key prognostic variables, including fibrosis stage, HBV virologic control, and prior interferon exposure. Furthermore, most available trials have relatively short treatment and follow‐up periods (often 48 weeks on therapy plus 24 weeks off‐treatment), making it difficult to assess the long‐term efficacy, especially after discontinuation of therapy, as relapse after treatment cessation is common, particularly after BLV monotherapy discontinuation. Most studies report surrogate endpoints, such as a decline in HDV RNA, rather than histologic improvement or definitive clinical outcomes. Limited real‐world data is available regarding the impact on fibrosis, decompensation, HCC, and survival.

Additionally, HBV‐related parameters were inconsistently reported across the included studies. Variations in measurement units, reference ranges, and reported biomarkers were observed, with some studies providing quantitative HBsAg levels whereas others reported HBV DNA levels. Posttreatment data for these markers were frequently unavailable. This heterogeneity and incomplete reporting precluded a formal meta‐analytic evaluation of HBV treatment response, particularly in studies assessing BLV‐containing regimens. A systematic assessment of HBV‐specific outcomes was beyond the predefined scope of this review.

Several of the smaller studies had open‐label designs and small sample sizes, which may overestimate treatment effects. Moreover, there is potential for publication bias, as negative or null studies may be underrepresented. Most published studies reported positive virologic outcomes with a relative paucity of negative or neutral studies in the literature. Smaller peginterferon cohort studies with poor HDV RNA suppression are rarely reported outside of older interferon‐era data. Additionally, the number of high‐quality randomized controlled trials remains limited, and some included studies may represent overlapping patient cohorts, potentially inflating effect estimates.

Future research should prioritize well‐powered, head‐to‐head randomized controlled trials comparing BLV monotherapy and combination therapy to determine the optimal regimen for both viral suppression and long‐term clinical improvement. There is a need for focus on newer HBV‐directed therapies that can cause a functional cure, clear HBsAg, which in turn stops HDV replication. Trials with longer posttreatment follow‐up, beyond 48 weeks, are needed to define stopping guidelines and evaluate whether sustained HDV RNA suppression translates into histologic improvement, fibrosis regression, and reduction in hepatic decompensation, HCC, and transplant‐free survival. Persistent HDV RNA suppression at follow‐up was minimal in the trials included in our study, necessitating further investigation into the off‐treatment durability of response and the benefit of maintenance therapy.

Moreover, postmarketing surveillance and large real‐world registries are needed as they can offer insight into the safety and cost‐effectiveness of these therapies. Real‐world data collection should also be expanded to better understand effectiveness in diverse populations, including patients with compensated or decompensated cirrhosis, HBV coinfection, and immunosuppressed patients.

Given the obligate relationship between HBV and HDV, future studies with standardized reporting of HBV biomarkers are necessary to better delineate potential interactions between HBV suppression and HDV treatment response. Finally, emerging therapies, including prenylation inhibitors such as lonafarnib [26] and entry inhibitors like HH003 [27], warrant further investigation as they provide optimism that combination regimens may eventually achieve functional cure or durable off‐treatment suppression. Future studies should explore combinations of antiviral and immune‐modulating agents to maximize virologic clearance, minimize relapse, and potentially eliminate the need for indefinite therapy.

This network meta‐analysis provides the most comprehensive synthesis to date comparing BLV, pegylated interferon, and NAs in the treatment of chronic HDV infection. Our findings reinforce that BLV, particularly when combined with pegylated interferon‐alpha, offers the greatest likelihood of achieving HDV RNA suppression at the EOT. However, sustained virologic responses remain limited, and improvements in biochemical and histological outcomes are inconsistent across regimens, highlighting the need for therapies that achieve durable viral control. The current evidence base is limited by heterogeneity in trial design, small sample sizes, and reliance on surrogate endpoints such as HDV RNA and ALT normalization. Therefore, future studies with standardized endpoints, longer follow‐up periods, and evaluation of clinically significant outcomes are needed. Until such data are available, management of chronic HDV should be individualized, balancing the potential benefits of viral suppression with the need for prolonged treatment, routine surveillance, and cost considerations.

## Funding

No funding was received for this manuscript.

## Conflicts of Interest

The authors declare no conflicts of interest.

## Data Availability

The data that support the findings of this study are available from the corresponding author upon reasonable request.
